# Marital status, household size, and lifestyle changes during the first COVID-19 pandemic: NIPPON DATA2010

**DOI:** 10.1371/journal.pone.0283430

**Published:** 2023-03-27

**Authors:** Makiko Abe, Hisatomi Arima, Atsushi Satoh, Nagako Okuda, Hirokazu Taniguchi, Nobuo Nishi, Aya Higashiyama, Harumitsu Suzuki, Aya Kadota, Takayoshi Ohkubo, Hirotsugu Ueshima, Katsuyuki Miura, Akira Okayama, for the NIPPON DATA2010 Research Group

**Affiliations:** 1 Department of Preventive Medicine and Public Health, Faculty of Medicine, Fukuoka University, Fukuoka, Japan; 2 Division of Applied Life Sciences, Graduate School of Life and Environmental Sciences, Kyoto Prefectural University, Kyoto, Japan; 3 International Center for Nutrition and Information, National Institute of Health and Nutrition, National Institutes of Biomedical Innovation, Health and Nutrition, Tokyo, Japan; 4 Department of Hygiene, Wakayama Medical University, Wakayama, Japan; 5 NCD Epidemiology Research Center, Shiga University of Medical Science, Otsu, Japan; 6 Department of Hygiene and Public Health, Teikyo University School of Medicine, Tokyo, Japan; 7 Research Institute of Strategy for Prevention, Tokyo, Japan; University Sains Malaysia, MALAYSIA

## Abstract

Stay-at-home strategies taken during the COVID-19 pandemic changed our lifestyle drastically. Although marital status and household size are important social determinants of health that affect lifestyle, their impacts on lifestyle during the pandemic are still unclear. We aimed to evaluate the association between marital status, household size, and lifestyle changes during the first pandemic in Japan. Questionnaire surveys on lifestyle changes from before to during the first COVID-19 pandemic were conducted on October 2020 in Japan. Classified into age groups, multivariable logistic regression analysis was performed to examine the combined association of marital status and household size on lifestyle, adjusted for potential confounders including socioeconomic factors. In our prospective cohort study, 1928 participants were included. Among older participants, the singles living alone were likely to perceive more unhealthy lifestyle changes (45.8%), compared with the married (33.2%), and significantly associated with at least one unhealthy change [adjusted odds ratio (OR): 1.81, 95% confidence interval (CI): 1,18–2.78], mainly due to decreased physical activity and increased alcohol consumption. Meanwhile, the younger participants showed no significant association between marital status, household size, and unhealthy changes, while those living alone had 2.87 times higher odds of weight gain (≥ 3 kg) than the married (adjusted OR: 2.87, 95% CI: 0.96–8.54) during the pandemic. Our findings suggest that older singles living alone are potentially vulnerable subgroups to drastic social changes which warrant special attention to prevent adverse health outcomes and additional burden on health systems in the following future.

## Introduction

Since the world’s first case of COVID-19 was discovered in 2019, global containment strategies to prevent the spread of COVID-19 have imposed unprecedented physical, mental, and social burdens. In Japan, more than 90% of schools suspended in-person classes on April 2020 [1], when the first state of emergency swept through the nation [2]. Accordingly, population mobility was reduced by more than 50% in metropolitan areas [3], and all industry activity index declined by 10% between December 2019 and May 2020, mainly due to the mass closure of the service sector including gyms, retail shops, and restaurants [4]. People were overwhelmed by the tremendous psychological stress of loneliness, boredom, fear, and stigma of infection, financial hardship, and severe supply shortages [5], and the long-lasting stress led to negative lifestyle changes, including binge eating, insomnia, and excessive alcohol consumption [5–7]. For example, weight gain caused by behavioral changes during the pandemic is well illustrated by the new-coined word “depreobesity” [8]. A meta-analysis [9] showed a significant increase in both body weight and body mass index (1.57 kg and 0.31 kg/m^2^, respectively) before and after the lockdown. In addition, a large French cohort study [10] reported a decrease in physical activity and consumption of fresh vegetables, and an increase in the consumption of alcohol and sweets during the lockdown. Furthermore, a cohort study conducted using online food ordering data in Singapore [11] showed a gradual increase in unhealthy food ordering, such as barbecue or fried food after the implementation of the lockdown.

Lifestyle is closely related to marital status and household size [12–14]; During the COVID-19 pandemic, singles perceived more loneliness and stress [15–17] and engaged in less physical activity than spousal pairs [18], while household size is reportedly associated with the volume of dietary intake, vegetable consumption, physical activity, and psychological stress [19–22]. However, few studies have examined the combined association of those two factors with lifestyle changes in the context of the COVID-19 pandemic. Therefore, we aimed to evaluate the combined association of marital status and household size with lifestyle changes during the first COVID-19 pandemic in Japan.

## Materials and methods

### Study design

This study is a prospective cohort study, where participants were recruited from cohort members of the National Integrated Project for Prospective Observation of Non-communicable Disease and its Trends in the Aged (NIPPON DATA2010) [23]. This project combined data from the National Health and Nutrition Survey (NHNS2010) [24] and the Comprehensive Survey of Living Conditions of the People on Health and Welfare (CSLC2010) [25]. These surveys were conducted by the Ministry of Health, Labour and Welfare of Japan. Briefly, in 2010, the CSLC was conducted to survey national living conditions in approximately 290,000 households from 5510 randomly selected areas throughout Japan. Of the 5510 areas, 300 areas were selected to conduct NHNS to survey lifestyles and collect blood samples in the same year, when trained interviewers obtained informed consent for NIPPON DATA2010. A total of 2898 participants (1239 men and 1659 women aged 20 years and older) in these areas agreed to participate in the baseline NIPPON DATA2010 survey. Of the participants, seven were excluded because it was impossible to merge the data. The remaining 2891 participants (1236 men and 1655 women) provided baseline data. We included 1928 participants aged 30 years and older as of 2020 in this study, after excluding participants who were lost to follow-up (n = 647) or who had data missing from the questionnaires or on marital status and household size at baseline (n = 316). The response rate was 93.4%.

### Ethics statement

We obtained written informed consent from all the participants. This study was approved by the Institutional Review Board of Shiga University of Medical Science (No. 22–29, 2010) and the Fukuoka University Clinical Research and Ethics Center (U21-09-001).

### Socioeconomic factors

Data on annual household income, employment status, years of education, and household size at baseline were obtained using self-administered questionnaires for NHNS2010, CSLC2010, and NIPPON DATA2010. Annual household income was classified into high (≥6 000 000 yen), middle (2 000 000–6 000 000 yen), and low (<2 000 000 yen), and employment status was defined as employed or self-employed, underemployed, or unemployed. Years of education were classified into three categories: <9, 9–12, and ≥13 years. Marital status and household size were categorized as married individuals or singles living with someone and singles living alone. The widowed or separated individuals were classified into a single group.

### Other factors

At baseline, when body weight and height were measured, participants wore light clothing without shoes. Each participant’s body mass index (BMI) was calculated by dividing their body weight in kilograms by height in meters squared. Smoking and drinking habits were classified into current, past/non-smoker, and drinker groups.

### Unhealthy changes in lifestyles during the COVID-19 pandemic

We distributed questionnaires on differences in body weight and lifestyle behaviors before and during the lockdown in Japan from April to May 2020, in October 2020 [2]. The topics covered by the questionnaires included changes in body weight (≥ +3 kg, +1 - +3 kg, -1 - +1 kg, -3 - -1 kg, or < - 3 kg), and frequency (increased, not changed, or decreased) of takeout food ordering, physical activity, eating between meals, and consumption of alcohol and vegetables from before to during the first COVID-19 pandemic in Japan. Physical activity included some activity indices, including sports, occupation, and housework. Unhealthy changes were defined as follows: 1) weight gain (≥ +3 kg), 2) an increase in frequency of eating between meals, or 3) alcohol consumption, 4) a decrease in frequency of vegetable consumption, or 5) physical activity, and 6) at least one unhealthy change mentioned above.

### Statistical analysis

The Kruskal–Wallis test was used to identify mean differences in age and BMI as continuous variables. For categorical variables, Fisher’s exact test was used to examine whether there were any differences in the observed distributions among the marital status and household size categories. Next, we stratified the number and percentage of unhealthy changes according to marital status and household size. We then performed a multivariate logistic regression analysis to examine whether marital status and household size were associated with unhealthy lifestyle changes after adjusting for covariates (age, sex, BMI, annual household income, employment status, years of education, and smoking and drinking habits at baseline). Socioeconomic factors were included in the model because they are also regarded as determinants of health from the perspective of affordability, accessibility, and availability of healthcare information and resources [26–29]. All analyses were performed by dividing the participants into two age groups with an age cut-off of 65 years. Statistical significance was set at P<0.05. All analyses were performed using Stata SE version 16 (StataCorp. 2019. Stata Statistical Software: Release 16. College Station, TX: StataCorp LLC.) and SAS (version 9.4; SAS Institute, Inc., Cary, NC).

## Results

Participants’ baseline characteristics are shown in Table 1. Women were more prevalent in all groups (married individuals: 57.0%, single people living with someone: 71.6%, single people living alone: 60.7%); both mean age and BMI were the highest among single people living alone (age: 62.4 years, BMI: 23.5 kg/m^2^). Single people living alone tended to be less educated, had lower household income, and were more likely to be unemployed at baseline than the other groups. More than half of the married individuals were current drinkers (55.0%), and there were no significant differences in smoking habits between the three groups.

**Table 1:** Baseline characteristics of participants according to marital status and household size.

	Marital status and household size			*P* value^a^
	Married individuals	Singles living with someone	Singles living alone	
**Aged 30 to 64 years, n**	569	145	44	
Age (years), mean (SD)	42.3 (7.7)	34.4 (10.4)	40.6 (9.7)	<0.001
BMI (kg/m²), mean (SD)	22.6 (3.5)	22.5 (4.3)	22.9 (3.1)	0.381
Sex, n (%)				<0.001
Men	202 (35.5)	51 (35.2)	29 (65.9)	
Women	367 (64.5)	94 (64.8)	15 (34.1)	
Household income (yen/year), n (%)				<0.001
≥6,000,000	207 (36.4)	30 (20.7)	4 (9.1)	
2,000,000–6,000,000	278 (48.9)	74 (51.0)	33 (75.0)	
<2,000,000	28 (4.9)	23 (15.9)	5 (11.4)	
Unknown	56 (9.8)	18 (12.4)	2 (4.6)	
Employment status, n (%)				0.004
Employed or self-employed	360 (63.3)	96 (66.2)	37 (84.1)	
Underemployed	63 (11.1)	14 (9.7)	7 (15.9)	
Unemployed	135 (23.7)	26 (17.9)	0 (0)	
Unknown	11 (1.9)	9 (6.2)	0 (0)	
Education (years), n (%)				0.007
≥13	316 (55.5)	88 (60.7)	27 (61.4)	
9–12	234 (41.1)	46 (31.7)	12 (27.3)	
<9	19 (3.3)	11 (7.6)	5 (11.4)	
Unknown	0 (0)	0 (0)	0 (0)	
Smoking habit, n (%)				0.594
Current smoker	108 (19.0)	30 (20.7)	11 (25.0)	
Past / non-smoker	460 (80.8)	115 (79.3)	33 (75.0)	
Unknown	1 (0.2)	0 (0)	0 (0)	
Drinking habit, n (%)				0.187
Current drinker	341 (59.9)	78 (53.8)	30 (68.2)	
Past / non-drinker	228 (40.1)	67 (46.2)	14 (31.8)	
Unknown	0 (0)	0 (0)	0 (0)	
**Aged ≥65 years, n**	955	73	142	
Age (years), mean (SD)	66.0 (6.7)	69.3 (9.0)	69.1 (7.3)	<0.001
BMI (kg/m²), mean (SD)	23.4 (3.0)	24.2 (3.6)	23.7 (3.7)	0.149
Sex, n (%)				<0.001
Men	453 (47.4)	11 (15.1)	44 (31.0)	
Women	502 (52.6)	62 (84.9)	98 (69.0)	
Household income (yen / year), n (%)				<0.001
≥6,000,000	156 (16.3)	10 (13.7)	3 (2.1)	
2,000,000–6,000,000	571 (59.8)	39 (53.4)	50 (35.2)	
<2,000,000	139 (14.6)	16 (21.9)	81 (57.0)	
Unknown	89 (9.3)	8 (11.0)	8 (5.6)	
Employment status, n (%)				0.477
Employed or self-employed	343 (35.9)	23 (31.5)	41 (28.9)	
Underemployed	68 (7.1)	6 (8.2)	6 (4.2)	
Unemployed	500 (52.4)	38 (52.1)	79 (55.6)	
Unknown	44 (4.6)	6 (8.2)	16 (11.3)	
Education (years), n (%)				0.002
≥13	212 (22.2)	9 (12.3)	31 (21.8)	
9–12	486 (50.9)	35 (48.0)	54 (38.0)	
<9	257 (26.9)	29 (39.7)	57 (40.1)	
Unknown	0 (0)	0 (0)	0 (0)	
Smoking habit, n (%)				0.459
Current smoker	116 (12.2)	6 (8.2)	13 (9.2)	
Past / non-smoker	835 (87.4)	67 (91.8)	128 (90.1)	
Unknown	4 (0.4)	0 (0)	1 (0.7)	
Drinking habit, n (%)				0.003
Current drinker	497 (52.0)	26 (35.6)	59 (41.6)	
Past / non-drinker	454 (47.5)	47 (64.4)	82 (57.8)	
Unknown	4 (0.4)	0 (0)	1 (0.7)	

BMI, body mass index; SD, standard deviation.

^a^*P* values were calculated using Kruskal–Wallis test for continuous variables and Fisher’s exact test for categorical variables excluding the unknown data.

Table 2 shows the number and percentages of participants who acquired unhealthy lifestyle changes. A decrease in the frequency of physical activity was the most common lifestyle change, ranging from 26% to 35%, and 30% to 40% of the participants had at least one unhealthy change across any group of marital status and household size. Among the older participants, singles living alone had more unhealthy lifestyle changes (*P* = 0.002). Meanwhile, among the younger ones, unhealthy lifestyle changes were more likely to be common among married individuals (*P* = 0.067).

**Table 2:** Percentages of subjects who acquired unhealthy lifestyle changes during the COVID-19 pandemic.

	Marital status and household size			*P* value^a^
	Married individuals	Singles living with someone	Singles living alone	
**Aged 30 to 64 years, n**	569	145	44	
The number of unhealthy lifestyle changes, n (%)				0.067
None	294 (51.7)	77 (53.1)	26 (59.1)	
1	165 (29.0)	37 (25.5)	14 (31.8)	
2	86 (15.1)	22 (15.2)	3 (6.8)	
3	5 (0.9)	5 (3.5)	1 (2.3)	
4	2 (0.4)	4 (2.8)	0 (0)	
5	1 (0.2)	0 (0)	0 (0)	
At least one unhealthy lifestyle change	275 (48.3)	68 (46.9)	18 (40.9)	
Unhealthy lifestyle changes, n (%)				0.011
Weight gain (≥3 kg)	35 (6.2)	16 (11.0)	5 (11.4)	
Increased frequency of eating between meals	109 (19.2)	31 (21.4)	1 (2.3)	
Decreased frequency of vegetable consumption	26 (4.6)	9 (6.2)	2 (4.6)	
Decreased frequency of physical activity	176 (30.9)	48 (33.1)	13 (29.6)	
Increased frequency of alcohol consumption	67 (11.8)	8 (5.5)	2 (4.6)	
**Aged ≥65 years, n**	955	73	142	
The number of unhealthy lifestyle changes, n (%)				0.002
None	638 (66.8)	41 (56.2)	77 (54.2)	
1	219 (22.9)	26 (35.6)	49 (34.5)	
2	72 (7.5)	4 (5.5)	12 (8.5)	
3	26 (2.7)	1 (1.4)	3 (2.1)	
4	0 (0)	1 (1.4)	1 (0.7)	
5	0 (0)	0 (0)	0 (0)	
At least one unhealthy lifestyle change	317 (33.2)	32 (43.8)	65 (45.8)	
Unhealthy lifestyle changes, n (%)				0.782
Weight gain (≥3 kg)	25 (2.6)	2 (2.7)	6 (4.2)	
Increased frequency of eating between meals	104 (10.9)	8 (11.0)	23 (16.2)	
Decreased frequency of vegetable consumption	55 (5.8)	5 (6.9)	12 (8.5)	
Decreased frequency of physical activity	233 (24.4)	26 (35.6)	40 (28.2)	
Increased frequency of alcohol consumption	24 (2.5)	0 (0)	5 (3.5)	

^a^*P* values were calculated using Fisher’s exact test.

The combined association of marital status and household size with lifestyle changes during the pandemic is shown in Table 3. Among older participants aged ≥65 years, compared with the married, singles living alone showed significantly higher odds of having unhealthy changes [odds ratio (OR): 1.81; 95% confidence interval (CI): 1.18–2.78, *P* = 0.007], which are mainly ascribed to eating between meals (OR: 1.64; 95% CI: 0.91–2.96, *P* = 0.097), drinking alcohol (OR: 2.84; 95% CI: 0.81–9.89, *P* = 0.102), and engaging in less physical activity (OR: 1.42; 95% CI: 0.89–1.27, *P* = 0.143). Regarding socioeconomic status, participants with higher household income and education showed more unhealthy lifestyle changes than those with lower ones [<2 000 000 yen/year in household income, OR: 0.60; 95% CI: 0.38–0.97, *P* = 0.037 (reference: ≥ 6 000 000 yen/year); <9 years of education: OR: 0.63; 95% CI: 0.43–0.94, *P* = 0.022 (reference: ≥13 years)]. Specifically, those with higher income and education were likely to have less frequency of physical activity [<2 000 000 yen/year, OR: 0.53; 95% CI: 0.32–0.88, *P* = 0.015 (reference: ≥6 000 000 yen/year); <9 years: OR: 0.59; 95% CI: 0.39–0.91, *P* = 0.017 (reference: ≥13 years)], and more frequency of alcohol consumption [<2 000 000 yen/year, OR: 0.25; 95% CI: 0.05–1.24, *P* = 0.091 (reference: ≥6 000 000 yen/year); <9 years, OR: 0.51; 95% CI: 0.15–1.78, *P* = 0.289 (reference: ≥13 years)].

**Table 3:** Associations between baseline socioeconomic status and unhealthy lifestyle changes during the COVID-19 pandemic.

	Aged 30 to 64 years		*P* value	Aged ≥65 years		*P* value
	n / total (%)^a^	Adjusted odds ratio (95% CI)^b^		n / total (%)^a^	Adjusted odds ratio (95% CI)^b^	
**Unhealthy lifestyle changes** ^c^						
Marital status and household size						
Married individuals	275/569 (51.7)	1.00 (reference)		317/955 (33.2)	1.00 (reference)	
Singles living with someone	68/145 (46.9)	0.97 (0.63–1.47)	0.876	32/73 (43.8)	1.63 (0.94–2.84)	0.083
Singles living alone	18/44 (40.9)	0.86 (0.44–1.70)	0.672	65/142 (45.8)	1.81 (1.18–2.78)	0.007
Household income (yen/year)						
≥6,000,000	123/241 (51.0)	1.00 (reference)		66/169 (39.1)	1.00 (reference)	
2,000,000–6,000,000	180/385 (46.8)	0.87 (0.62–1.22)	0.421	235/660 (35.6)	0.78 (0.54–1.13)	0.190
<2,000,000	26/56 (46.4)	0.84 (0.44–1.61)	0.597	80/236 (33.9)	0.60 (0.38–0.97)	0.037
Employment status						
Employed or self-employed	223/493 (45.2)	1.00 (reference)		127/407 (31.2)	1.00 (reference)	
Underemployed	41/84 (48.8)	0.84 (0.50–1.42)	0.522	31/80 (38.8)	1.20 (0.71–2.05)	0.493
Unemployed	84/161 (52.2)	1.35 (0.89–2.05)	0.154	226/617 (36.6)	1.19 (0.88–1.59)	0.256
Education (years)						
≥13	216/431 (50.1)	1.00 (reference)		102/252 (40.5)	1.00 (reference)	
9–12	129/292 (44.2)	0.75 (0.53–1.04)	0.083	204/575 (35.5)	0.80 (0.57–1.12)	0.194
<9	16/35 (45.7)	0.77 (0.34–1.77)	0.538	108/343 (31.5)	0.63 (0.43–0.94)	0.022
**Weight gain (≥ 3 kg)**						
Marital status and household size						
Married individuals	35/569 (6.2)	1.00 (reference)		25/955 (2.6)	1.00 (reference)	
Singles living with someone	16/145 (11.0)	2.93 (1.45–5.90)	0.003	2/73 (2.7)	1.15 (0.25–5.25)	0.856
Singles living alone	5/44 (11.4)	2.87 (0.96–8.54)	0.058	6/142 (4.2)	1.84 (0.61–5.55)	0.279
Household income (yen/year)						
≥6,000,000	20/241 (8.3)	1.00 (reference)		6/169 (3.6)	1.00 (reference)	
2,000,000–6,000,000	29/385 (7.5)	0.67 (0.35–1.28)	0.223	17/660 (2.6)	0.61 (0.23–1.62)	0.320
<2,000,000	3/56 (5.4)	0.24 (0.05–1.16)	0.076	6/236 (2.5)	0.53 (0.15–1.90)	0.328
Employment status						
Employed or self-employed	39/493 (7.9)	1.00 (reference)		11/407 (2.7)	1.00 (reference)	
Underemployed	6/84 (7.1)	1.19 (0.46–3.09)	0.724	3/80 (3.8)	1.68 (0.43–6.52)	0.456
Unemployed	9/161 (5.6)	1.09 (0.47–2.51)	0.842	18/617 (2.9)	1.24 (0.52–2.96)	0.627
Education (years)						
≥13	31/431 (7.2)	1.00 (reference)		5/252 (2.0)	1.00 (reference)	
9–12	23/292 (7.9)	1.03 (0.54–1.94)	0.934	17/575 (3.0)	1.62 (0.53–4.99)	0.856
<9	2/35 (5.7)	0.67 (0.13–3.44)	0.627	11/343 (3.2)	1.43 (0.41–5.03)	0.576
**Increased frequency of eating between meals**						
Marital status and household size						
Married individuals	109/569 (19.2)	1.00 (reference)		104/955 (10.9)	1.00 (reference)	
Singles living with someone	31/145 (21.4)	1.22 (0.73–2.03)	0.446	8/73 (11.0)	0.80 (0.33–1.96)	0.628
Singles living alone	1/44 (2.3)	0.14 (0.02–1.04)	0.054	23/142 (16.2)	1.64 (0.91–2.96)	0.097
Household income (yen/year)						
≥6,000,000	48/241 (19.9)	1.00 (reference)		20/169 (11.8)	1.00 (reference)	
2,000,000–6,000,000	72/385 (18.7)	0.97 (0.63–1.49)	0.887	79/660 (12.0)	1.00 (0.57–1.73)	0.987
<2,000,000	12/56 (21.4)	1.12 (0.51–2.49)	0.774	25/236 (10.6)	0.89 (0.44–1.81)	0.746
Employment status						
Employed or self-employed	80/493 (16.2)	1.00 (reference)		48/407 (11.8)	1.00 (reference)	
Underemployed	14/84 (16.7)	0.80 (0.40–1.60)	0.528	13/80 (16.3)	1.17 (0.57–2.42)	0.672
Unemployed	42/161 (26.1)	1.41 (0.88–2.27)	0.153	67/617 (10.9)	0.72 (0.47–1.11)	0.135
Education (years)						
≥13	82/431 (19.0)	1.00 (reference)		32/252 (12.7)	1.00 (reference)	
9–12	54/292 (18.5)	1.05 (0.69–1.60)	0.811	76/575 (13.2)	0.90 (0.56–1.46)	0.679
<9	5/35 (14.3)	0.79 (0.26–2.37)	0.672	27/343 (7.9)	0.56 (0.30–1.03)	0.062
**Decreased frequency of eating vegetables**						
Marital status and household size						
Married individuals	26/569 (4.6)	1.00 (reference)		55/955 (5.8)	1.00 (reference)	
Singles living with someone	9/145 (6.2)	1.24 (0.51–3.00)	0.638	5/73 (6.9)	1.41 (0.52–3.84)	0.503
Singles living alone	2/44 (4.6)	0.34 (0.04–3.00)	0.330	12/142 (8.5)	0.80 (0.33–1.95)	0.625
Household income (yen/year)						
≥6,000,000	10/241 (4.2)	1.00 (reference)		6/169 (3.6)	1.00 (reference)	
2,000,000–6,000,000	15/385 (3.9)	0.89 (0.38–3.94)	0.791	36/660 (5.5)	1.32 (0.54–3.26)	0.543
<2,000,000	9/56 (16.1)	2.85 (0.88–9.15)	0.079	22/236 (9.3)	2.18 (0.79–6.01)	0.133
Employment status						
Employed or self-employed	23/493 (4.7)	1.00 (reference)		15/407 (3.7)	1.00 (reference)	
Underemployed	4/84 (4.8)	1.22 (0.38–3.94)	0.738	8/80 (10.0)	2.42 (0.88–6.65)	0.087
Unemployed	10/161 (6.2)	1.45 (0.59–3.55)	0.417	40/617 (6.5)	1.65 (0.85–3.22)	0.141
Education (years)						
≥13	18/431 (4.2)	1.00 (reference)		17/252 (6.8)	1.00 (reference)	
9–12	13/292 (4.5)	0.77 (0.34–1.76)	0.539	31/575 (5.4)	0.67 (0.33–1.33)	0.253
<9	6/35 (17.1)	2.28 (0.64–8.17)	0.204	24/343 (7.0)	0.69 (0.32–1.50)	0.350
**Decreased frequency of physical activity**						
Marital status and household size						
Married individuals	176/569 (30.9)	1.00 (reference)		233/955 (24.4)	1.00 (reference)	
Singles living with someone	48/145 (33.1)	1.17 (0.74–1.84)	0.499	26/73 (35.6)	1.82 (1.02–3.23)	0.041
Singles living alone	13/44 (29.6)	1.35 (0.65–2.78)	0.423	40/142 (28.2)	1.42 (0.89–1.27)	0.143
Household income (yen/year)						
≥6,000,000	93/241 (38.6)	1.00 (reference)		54/169 (32.0)	1.00 (reference)	
2,000,000–6,000,000	111/385 (28.8)	0.65 (0.45–0.93)	0.019	168/660 (25.5)	0.69 (0.47–1.02)	0.062
<2,000,000	11/56 (19.6)	0.40 (0.19–0.87)	0.020	55/236 (23.3)	0.53 (0.32–0.88)	0.015
Employment status						
Employed or self-employed	145/493 (29.4)	1.00 (reference)		85/407 (20.9)	1.00 (reference)	
Underemployed	27/84 (32.1)	0.82 (0.46–1.47)	0.512	19/80 (23.8)	1.15 (0.63–2.09)	0.646
Unemployed	58/161 (36.0)	1.37 (0.88–2.12)	0.160	174/617 (28.2)	1.42 (1.03–1.96)	0.033
Education (years)						
≥13	149/431 (34.6)	1.00 (reference)		77/252 (30.6)	1.00 (reference)	
9–12	80/292 (27.4)	0.71 (0.49–1.02)	0.061	150/575 (26.1)	0.81 (0.56–1.16)	0.244
<9	8/35 (22.9)	0.61 (0.23–1.65)	0.330	72/343 (21.0)	0.59 (0.39–0.91)	0.017
**Increased frequency of alcohol consumption**						
Marital status and household size						
Married individuals	67/569 (11.8)	1.00 (reference)		24/955 (2.5)	1.00 (reference)	
Singles living with someone	8/145 (5.5)	0.34 (0.13–0.89)	0.027	0/73 (0)	NE^d^	NE^d^
Singles living alone	2/44 (4.6)	0.35 (0.08–1.56)	0.169	5/142 (3.5)	2.84 (0.81–9.89)	0.102
Household income (yen/year)						
≥6,000,000	31/241 (12.9)	1.00 (reference)		9/169 (5.3)	1.00 (reference)	
2,000,000–6,000,000	32/385 (8.3)	0.84 (0.48–1.48)	0.549	13/660 (2.0)	0.34 (0.12–0.92)	0.034
<2,000,000	5/56 (8.9)	0.30 (0.03–2.72)	0.287	4/236 (1.7)	0.25 (0.05–1.24)	0.091
Employment status						
Employed or self-employed	55/493 (11.2)	1.00 (reference)		14/407 (3.4)	1.00 (reference)	
Underemployed	7/84 (8.3)	1.04 (0.40–2.70)	0.940	5/80 (6.3)	3.01 (0.95–9.54)	0.062
Unemployed	14/161 (8.7)	1.46 (0.69–3.07)	0.319	7/617 (1.1)	0.40 (0.13–1.18)	0.096
Education (years)						
≥13	45/431 (10.4)	1.00 (reference)		11/252 (4.4)	1.00 (reference)	
9–12	30/292 (10.3)	0.84 (0.48–1.48)	0.549	12/575 (2.1)	0.47 (0.18–1.22)	0.121
<9	2/35 (5.7)	0.30 (0.03–2.72)	0.287	6/343 (1.8)	0.51 (0.15–1.78)	0.289

CI, confidence interval.

^a^Unknown data were excluded.

^b^Odds ratios were calculated using logistic regression models adjusted for age, sex, body mass index, smoking habit, drinking habit, and socioeconomic factors at baseline.

^c^At least one unhealthy lifestyle change.

^d^Not estimable.

Meanwhile, among the younger participants, there was no significant association between marital status, household size, and unhealthy lifestyle changes (singles living alone, OR: 0.86; 95% CI: 0.44–1.70, *P* = 0.672). Interestingly, more single people experienced a weight gain of ≥3 kg than the married (single people living with someone, OR: 2.93; 95% CI: 1.45–5.90, *P* = 0.003; single people living alone, OR: 2.87; 95% CI: 0.96–8.54, *P* = 0.054). The inverse association between income and frequency of physical activity was also observed among the younger group [<2 000 000 yen/year in household income: OR: 0.40; 95% CI: 0.19–0.87, *P* = 0.020 (reference: ≥6 000 000 yen/year)].

## Discussion

Our study revealed that marital status and household size were significant effect modifiers of the association between the first COVID-19 pandemic and unhealthy lifestyle changes in Japan, irrespective of socioeconomic status. Social isolation and home confinement to prevent the spread of COVID-19 have drastically changed many aspects of our lifestyles, albeit without legally binding obligations. The magnitude of unhealthy lifestyle changes was significantly different across marital status and household size, and single older adults living alone were 1.8 times more likely to have at least one negative change.

### The unhealthy changes in lifestyles among older people living alone

Older adults living alone showed higher odds of having experienced at least one negative lifestyle change than married people. To our knowledge, no study has examined the association between unhealthy lifestyle changes during the pandemic, marital status, and household size among older adults. According to Lehtisalo et al. [22] and Salman et al. [18], older people living alone were more likely to experience decreased physical activity, decreased vegetable consumption, and increased loneliness than those living with someone during the lockdown in 2020. Although the authors did not assess the impacts of marital status on the outcomes, these findings would reflect the impacts of older adults living alone, since global data show that more than 90% of all one-person households are unmarried in developed countries [30].

Although social isolation does not necessarily parallel to emotional loneliness, Tilburg [31] documented people living alone manifested significantly more emotional loneliness than those who living with a spouse or partner. Hearne [19] also showed singles living alone were at a higher risk of psychological distress than those married and co-habiting during the lockdown. Loneliness and low levels of life satisfaction induce decreased physical activity [18], increased alcohol consumption [32] and emotional eating, which includes overeating of high-calorie foods or snacks to mitigate psychological stress [20]. Meanwhile, Poelman et al. [33] and Birditt et al. [17] documented that older people were less likely to perceive lockdown-related stress or change their lifestyles than younger people because they generally have fewer chances to face abrupt financial crisis due to the subsequent extreme psychological stress. However, these findings may have been modified by marital status and household size.

Over the decades, Japan has been facing super-aging challenges; the population aged ≥ 65 was approximately 28.8% (36 million) in 2020 and is expected to increase to 35% (39 million) by 2040 [34]. In addition, the percentage of one-person households among older people has doubled (27%) over 30 years [34, 35]. Decreased physical activity, unhealthy eating, and psychological stress predispose older people to impaired cognitive function and immune system [36, 37], sarcopenia [38], and obesity [20], which will impose a considerable burden of medical costs capacity even after convergence of COVID-19 pandemic. Furthermore, missing healthcare visits, decreased health care services, and highly recommended cocooning due to their vulnerability to infection exacerbated pre-existing chronic health conditions, including obesity, hypertension, and diabetes, which globally spread alongside the COVID-19 pandemic [39]. To tackle this problem, social cohesion and digital inclusion for older people living alone are crucial. Developing an age-friendly digital platform allows them to keep communication channels open to maintain healthcare intervention and social cohesion, including online exercise, medical appointments, and interaction with other people, keeping safety amid the infectious disease crises. Notably, Fingerman et al. [40] suggested older people living alone are more reactive than those living with someone to social contact with friends or care service providers.

### Socioeconomic factors and unhealthy changes in lifestyles

We found that higher income and education were likely to associate with unhealthy lifestyle changes, especially weight gain of more than 3 kg, less physical activity, and more alcohol consumption. One plausible explanation is the prevalence of teleworking, the rate of which almost tripled among Japanese employees during the first lockdown in 2020 [41]. Larger companies are more likely to offer to telework [42], which causes prolonged stay-at-home periods, and subsequently decreases the frequency of physical activity [43, 44]. In this sense, the implementation of teleworking may also underlie the association between higher education and unhealthy changes. The association between older people with higher income and more alcohol consumption might be reasoned by more disposable money to spend on alcohol. In fact, a multi-cohort study of the UK [45] demonstrated decreased alcohol consumption in the younger generation, who had more financial difficulties than the older generation during the lockdown. However, younger people with higher incomes did not show a significant increase in alcohol consumption compared to those with lower incomes. Some epidemiological studies reported that younger people appeared to prefer on-site drinking to drinking at home; therefore a sweeping closure of pubs and bars during the pandemic might not have led to increased alcohol consumption at home among younger people [32, 45].

### Weight gain during the COVID-19 pandemic

The younger singles had higher odds of weight gain (≥ 3 kg) than the married ones during the pandemic. While previous studies using online surveys showed no significant association between marital status and weight gain during lockdown [46–48], those who live in smaller households have been shown to be associated with less physical activity [22, 49], poorer sleep quality [50], and more psychological stress [19, 22, 51], leading to weight gain. Percentages of those who regularly work out in gyms or outside might have affected the association because of the closure of sports facilities [52].

The strengths of our study are the large sample size, high response rate, and wide age range of participants in the nation-wide survey. Quite a few surveys on lifestyle changes during the COVID-19 pandemic offered online questionnaires, where respondents are likely to be biased toward younger generations. Nonetheless, we have to mention several limitations. First, owing to the nature of a survey using questionnaires, we cannot eliminate the possibility of recall bias and attrition bias. Second, the percentages of essential workers, those in healthcare, delivery services, agriculture, and retail establishments might have affected our results, since they had fewer opportunities to stay at home, which would have potentially induced behavioral changes during the pandemic [53]. Third, methodological differences, including the duration of the research period, sample selection, and types of variables included in the quantitative analyses, may have caused discrepancies across studies. Fourth, there is a possibility that selection bias limited the internal validity of our study. Fifth, there might be changes in marital status and household size during the study period. However, a recent study reported both the percentages of marriage and divorce decreased by 10–20% during the pandemic possibly due to the stay-at-home recommendation in Japan [54]. Additionally, non-differential misclassification like this generally leads to a null hypothesis [55], however, we identified the significant association between marital status, household size, and unhealthy changes, as well as age effects. Although the household income of participants also may have changed from the baseline, the impacts would be trivial since over ten years the average annual change rates in household income is approximately 4%, which is hardly expected to change the categories participants belong. Lastly, we did not ask the content of foods or estimate the intake of nutrients using food frequency questionnaires to define unhealthy eating habits.

## Conclusion

We highlight that marital status and household size are important effect modifiers during the COVID-19 pandemic. Of note, older adults living alone are particularly vulnerable to drastic social changes, which warrant special attention to prevent adverse health outcomes.
